# A benchmark dataset of narrative student essays with multi-competency grades for automatic essay scoring in Brazilian Portuguese

**DOI:** 10.1016/j.dib.2025.111526

**Published:** 2025-03-27

**Authors:** Hilário Oliveira, Rafael Ferreira Mello, Péricles Miranda, Hyan Batista, Moésio Wenceslau da Silva Filho, Thiago Cordeiro, Ig Ibert Bittencourt, Seiji Isotani

**Affiliations:** a Federal Institute of Education Science and Technology of Espírito Santo, Serra, Espírito Santo, Brazil; b Federal Rural University of Pernambuco, Recife, Pernambuco, Brazil; c CESAR School, Recife, Pernambuco, Brazil; d Federal University of Alagoas, Maceió, Alagoas, Brazil; e University of São Paulo, São Carlos, São Paulo, Brazil; f Harvard Graduate School of Education, Cambridge, Massachusetts, USA

**Keywords:** Automated essay scoring, Elementary school, Narrative student essays, Artificial intelligence

## Abstract

This paper describes the development of a new database comprising 1235 narrative essays written in Portuguese by 5th-grade students in Brazil. The corpus construction process involved three main steps: acquiring and transcribing photos of the essays, annotating them based on a real pre-defined correction rubric by experts considering four key writing competencies (formal language use, textual typology, thematic coherence, and textual cohesion), and resolving disagreements between the annotators. Two human experts manually evaluated each essay using a five-point scale (Level I: Complete lack of domain - Level V: Excellent mastery) aligned with the correction rubric. In cases of disagreement between the initial evaluators, a third expert facilitated the divergences resolution. To the best of our knowledge, this is the first publicly available dataset of elementary school essays in Brazilian Portuguese that features narrative writing samples with corresponding grades across multiple competencies commonly used in writing assessment. We believe this resource can contribute to developing automatic essay scoring systems tailored for evaluating narrative texts written in Brazilian Portuguese.

Specifications Table

**Table:** 

Subject	Computer Science and Artificial intelligence
Specific subject area	Natural Language Processing, Machine learning and Automatic Essay Scoring (AES)
Type of data	Raw, analyzed, and filtered student's essay texts and their grades per competence.
Data collection	The dataset comprises essays manually written by 5th-year elementary school students from Brazil's public education system. Students were guided to compose a narrative essay inspired by a given prompt. Each essay was subsequently digitized and anonymized. Three human evaluators examined the texts, evaluating various competencies according to a predefined scoring rubric.
Data source location	The essays were collected at an elementary school in Brazil.
Data accessibility	Repository name: Brazilian Portuguese Narrative Essays Dataset Data identification number: 10.34740/kaggle/ds/4464018 Direct URL to data: 10.34740/kaggle/ds/4464018

## 1 Value of the Data

• This dataset [1] contains 1235 essays written in Portuguese by students from 5th year of elementary school in Brazil. Several annotations were made for each essay based on real correction rubrics defined by education specialists. To our knowledge, this is the first dataset of elementary school essays in Brazilian Portuguese for AES. The essays were collected at an elementary school in Brazil.
• Portuguese is a morphologically rich language spoken by more than 200 million people worldwide. Despite this, it is still a low-resource language in the Natural Language Processing (NLP) community. Directly adopting solutions proposed in other languages, such as English, is not always possible, especially in applications that require deeper linguistic analysis. This dataset seeks to fill this gap to boost the development of AES solutions.
• The development of new AES databases is crucial for advancing research, particularly for the Portuguese language, which remains underrepresented in this field. The proposed corpus provides a valuable resource for training and refining AES models tailored to Brazilian Portuguese, with expert-annotated grades across key writing competencies. By enabling researchers to enhance scoring methods and address challenges like bias and generalization, this dataset can support the creation of more accurate and fair AES systems. As these systems improve, they can be integrated into classrooms, helping teachers assess student writing more efficiently while providing timely, data-driven feedback. Ultimately, the continued expansion of AES databases in Portuguese will bridge the gap between research and real-world applications, fostering a more effective and equitable education system.
• This dataset can benefit NLP and Machine Learning (ML) researchers in developing new approaches for AES, specially designed for Brazilian Portuguese. The development of resources such as the proposed corpus enables the investigation of new algorithms, including neural network architectures [2], large language models [3], evolutionary algorithms [4], and others.
• The constructed corpus was successfully used in a competition involving several AES systems at the 16th International Conference on Computational Processing of Portuguese (PROPOR 2024). The results are available in [5].

## 2 Background

Automated Essay Scoring (AES) is a research area dedicated to developing computer systems that automatically evaluate the quality of written textual productions (essays) based on a pre-defined correction rubric [6,9]. AES systems can be a valuable resource for modernizing the essay grading process by offering an efficient and objective approach to assessing writing skills on a large scale [7]. The growing demand for effective assessment methods in educational contexts, where manual correction becomes unfeasible due to the volume of essays to be assessed, highlights the importance of developing more efficient solutions [6]. In addition to providing an agile response to large volumes of essays, AES systems offer the opportunity to implement immediate feedback, aiding the learning process and promoting the ongoing development of students’ writing skills.

The development of AES began with systems based on heuristics and hand-crafted rules, which assessed text quality using predefined linguistic features aligned with grading rubrics [9]. Over time, research shifted toward machine learning algorithms, requiring annotated datasets to train models to learn scoring patterns more effectively. This type of approach required extracting features from essays and developing labelled datasets to enable the training and evaluation of new models. More recently, with the rise of neural language models based on Deep Learning (DL) algorithms, the need for high-quality annotated data has become even greater, as these models rely on extensive datasets to achieve high accuracy and reliability [3]. As a result, creating comprehensive AES datasets has become essential for advancing the field, paving the way for more precise and adaptable automated grading systems that can better support real-world educational needs.

Despite the extensive research on AES, particularly for languages like English, studies focusing on Brazilian Portuguese are relatively scarce [8]. Existing research in Portuguese primarily focuses on evaluating dissertative-argumentative essays [2,8,10], especially considering textual productions in the context of the Brazilian National High School Exam (ENEM) [[11], [12], [13]].

## 3 Data Description

**Fig. 1:**
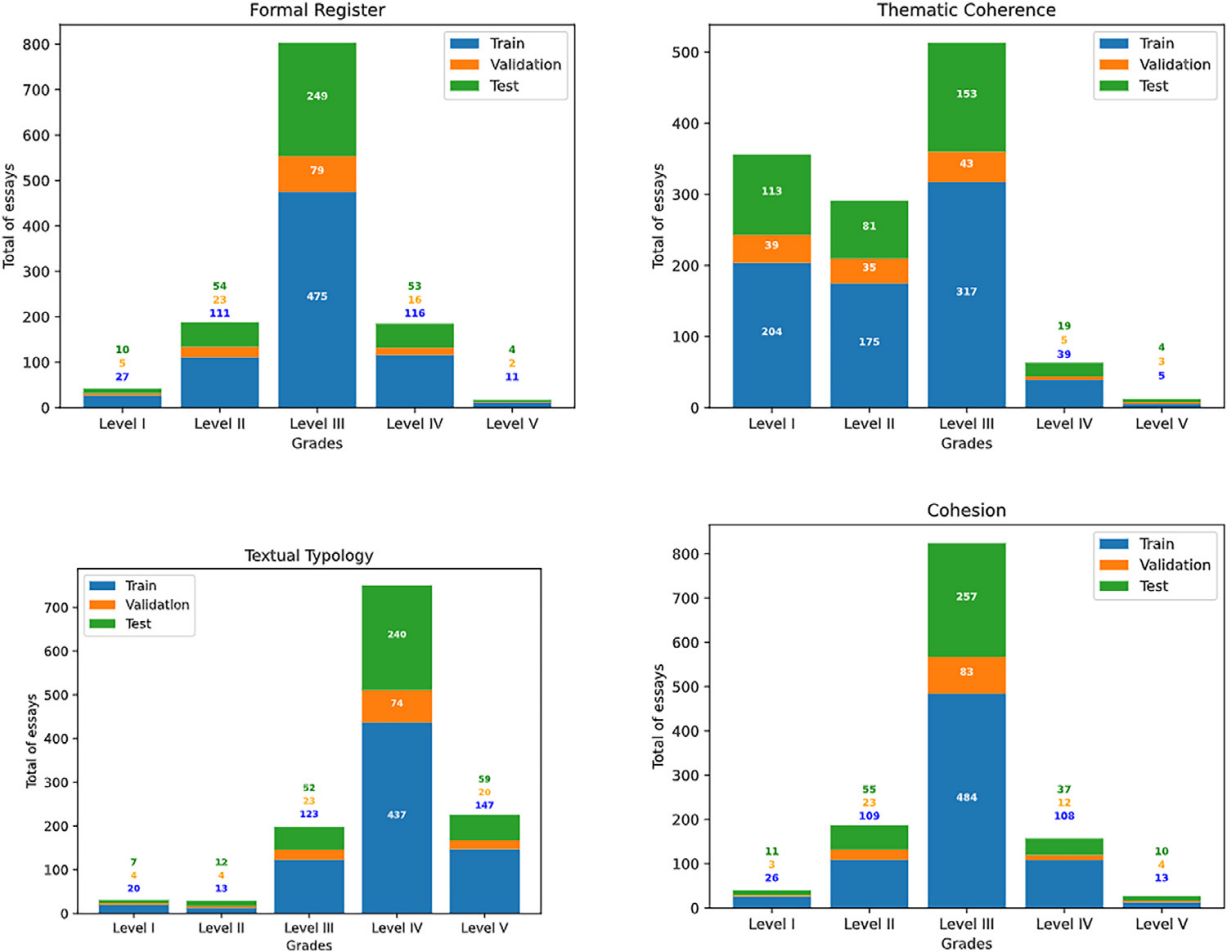
Essays distribution by level for each assessed competency on the train, validation, and test sets.

The dataset is partitioned into three subsets according to the following distribution: 60% (740) for training, 10% (125) for validation, and 30% (370) for testing. The essays are stored in Comma-Separated Value (CSV) file format, named *train.csv, validation.csv*, and *test.csv*, respectively. Each essay comprises the following fields: **(i) id**, serving as a unique identifier; **(ii) essay**, representing the raw text of the digitized writing; **(iii) prompt**, depicting the contextual situation (motivating text) for the narrative essay; and **(iv)** four columns containing scores for each of the four competencies (**formal_registration, thematic_coherence, narrative_rhetorical_structure, cohesion**). Each competency is assessed on an integer scale from 1 to 5, where higher scores reflect superior text quality and language proficiency, while lower scores indicate limited proficiency. Fig. 1 presents the distribution of essays across the subsets and the four evaluated competencies.

Table 1 presents an example of an essay randomly selected from the training set. This example includes the prompt that guides the student's writing, the essay itself, and the four grades assigned to each assessed skill.

**Table 1:** Example of an essay (in Portuguese) selected from the training set.

Field	Content
ID	2
Prompt	Chovia muito naquele dia, com trovões muito altos, vindos do céu. E depois que a chuva passou, encontrei no quintal da minha casa uma pedra muito brilhante ...
Essay	Chovia muito naquele dia, com trovões muito altos, vindos do céu. E depois que a chuva passou encontrei no quintal da minha cada uma pedra muito gigante brilhante não acreditei no que estava vendo, estava encantada com aquilo peguei e fui lá para dentro sem acreditar e guardei em um cofre, sem saber se realmente era real.
Formal Registration	4
Thematic Coherence	3
Narrative Rhetorical Structure	3
Cohesion	4

Some descriptive statistics related to the essay texts for train, validation, and test sets are shown in Table 2. The statistics computed were the total number of sentences and words per essay, the average and Standard Deviation (SD) of the total number of sentences and words per essay, and the average and SD of the total number of words per sentence. To compute the statistics, the essays were preprocessed using the spaCy^1^ toolkit. It can be seen by analyzing the standard deviation that there is great variability in the size (average of sentences and words) of the essays. As a rigid minimum and maximum essay length was not specified in the task rubric, students could write from succinct essays to larger texts.

**Table 2:** Descriptive statistics of the train, validation, and test sets.

Statistics	Train	Validation	Test
Total of Sentences	5235	974	2448
Total of Tokens	110,915	17,622	56,323
Average (SD) Sentences per Essay	7.07 (7.12)	7.79 (7.32)	6.61 (5.71)
Average (SD) Tokens per Essay	149.89 (69.75)	140.98 (62.64)	152.22 (70.11)
Average (SD) Tokens per Sentence	21.22 (28.95)	18.09 (24.09)	23.01 (31.28)

Table 3 compares the proposed dataset to other corpora commonly adopted in the literature for AES in Brazilian Portuguese. The other databases focus on high school, with the ENEM exam as the application domain. Although our corpus contains fewer essays than other corpora, it is important to highlight that, unlike them, each essay was manually evaluated by at least two human examiners.

**Table 3:** Statistics from other databases for Brazilian Portuguese AES compared with the proposed corpus.

Dataset	Context	Essays
Amorim and Veloso [14]	Dissertative-argumentative	1840
Marinho et al. [11]	Dissertative-argumentative	4572
Marinho et al. [12]	Dissertative-argumentative	6579
Silveira, Barbosa and Mauá [13]	Dissertative-argumentative	3586
Our dataset	Narrative	1235

## 4 Experimental Design, Materials and Methods

The proposed dataset was created as part of a broader project developed in partnership with the Ministry of Education of Brazil. In its latest version, it consists of 1235 narrative essays written in Portuguese by 5th-grade students, typically aged 10–14. The students were instructed to write a narrative text in Brazilian Portuguese, continuing a fictional story based on a given prompt, as illustrated in Table 1. The objective of the task was for students to extend the story by introducing new plot elements, describing scenarios, and developing characters, among other aspects. The prompts used in the corpus construction were selected because they were the same ones used by teachers in classroom activities during the data collection period.

The corpus construction process was carried out in three steps: Collection, annotation, and resolution of divergencies. The teachers took photos of the students’ essays in the data collection stage. Afterward, these essays were transcribed from images to texts. Two human experts independently analyzed the essays based on the following competencies (Formal register, Thematic coherence, Narrative textual typology, and Textual cohesion) adopted by the Center for Public Policy and Evaluation of Education in Brazil. Each essay has individual grades for each of the four competencies. It follows a brief description of these competencies:

1. **Formal Register**: This competency assesses the proper use of formal Brazilian Portuguese. It takes into account factors such as spelling errors, incorrect nominal and verbal agreement, improper nominal and verbal regency, and inappropriate use of punctuation marks.
2. **Thematic Coherence**: It evaluates the appropriate understanding of the writing prompt and its development, incorporating knowledge from different domains as required. It also assesses the plausibility of the produced text in relation to the given prompt.
3. **Narrative Rhetorical Structure (Textual Typology)**: This competency evaluates the essay's alignment with the narrative textual typology, considering the logical and sequential articulation of ideas, facts, and information. Additionally, the essay must include essential elements of this structure, such as a narrator, setting, temporal organization, and one or multiple characters engaging in actions.
4. **Textual Cohesion**: It assesses the proper use of linguistic mechanisms to establish connections between different elements of the text, including words, sentences, and paragraphs.

Table 4 presents the criteria adopted by the human evaluators for assigning each level of the correction rubric for every assessed competency. The criteria present aspects of the essay that the teachers need to analyze during the assessment to assign the appropriate level for each competence. Although the rubric correction criteria are well-defined, a degree of subjectivity remains, highlighting the complexity of this task for teachers.

**Table 4:** Correction criteria for every competence and each grade level.

Competence	Level I	Level II	Level III	Level IV	Level V
Formal Register	It exhibits a limited morphosyntactic structure with frequent deviations or predominantly syllabic-alphabetic writing.	It presents a scarce morphosyntactic structure, but with rarely recurring deviations.	It presents a consistent morphosyntactic structure with the recurrence of one type of deviation.	It presents a consistent morphosyntactic structure with punctual deviations.	It presents a well-used morphosyntactic structure with, at most, five punctual and non-recurrent deviations.

Thematic Coherence	It presents a tangent to the theme with/without coherent textual progression.	It exhibits inadequate textual progression, relying solely on the motivating situation's ideas or achieving full progression, but mainly through copied excerpts.	It exhibits complete textual progression but relies on paraphrases of the motivating situation or predictable ideas (common sense) that lack detail.	It presents complete textual progression, appropriating the motivating situation, based on predictable ideas, but with details.	It presents complete textual progression with a consistent repertoire that goes beyond the motivating situation.

Narrative Rhetorical Structure	It contains only descriptions of narrative elements (characters, narrator, temporal organization, settings) or predominantly features other textual styles.	It develops only one structuring part of the narrative plot (orientation, complication, or outcome) and/or includes just one narrative element.	It develops only two structuring parts of the narrative plot or presents all three but fails to develop two. Alternatively, it includes just two narrative elements.	It develops only two structuring parts of the narrative plot or presents all three but fails to develop two. Alternatively, it includes just two narrative elements.	It fully develops all structuring parts of the narrative plot and includes all essential narrative elements.

Textual Cohesion	Words and sentences are juxtaposed and disconnected, lacking articulation. However, cohesion is present through logical relationships or a limited cohesive repertoire with frequent deviations.	Scarce cohesive repertoire, but with specific deviations.	Low diversified cohesive repertoire with recurrence of one type of deviation.	Diverse cohesive repertoire with specific deviations that still affect the intelligibility of part of the text.	Diverse cohesive repertoire with the possibility of rare inadequacies that do not interfere with textual intelligibility.

**Fig. 2:**
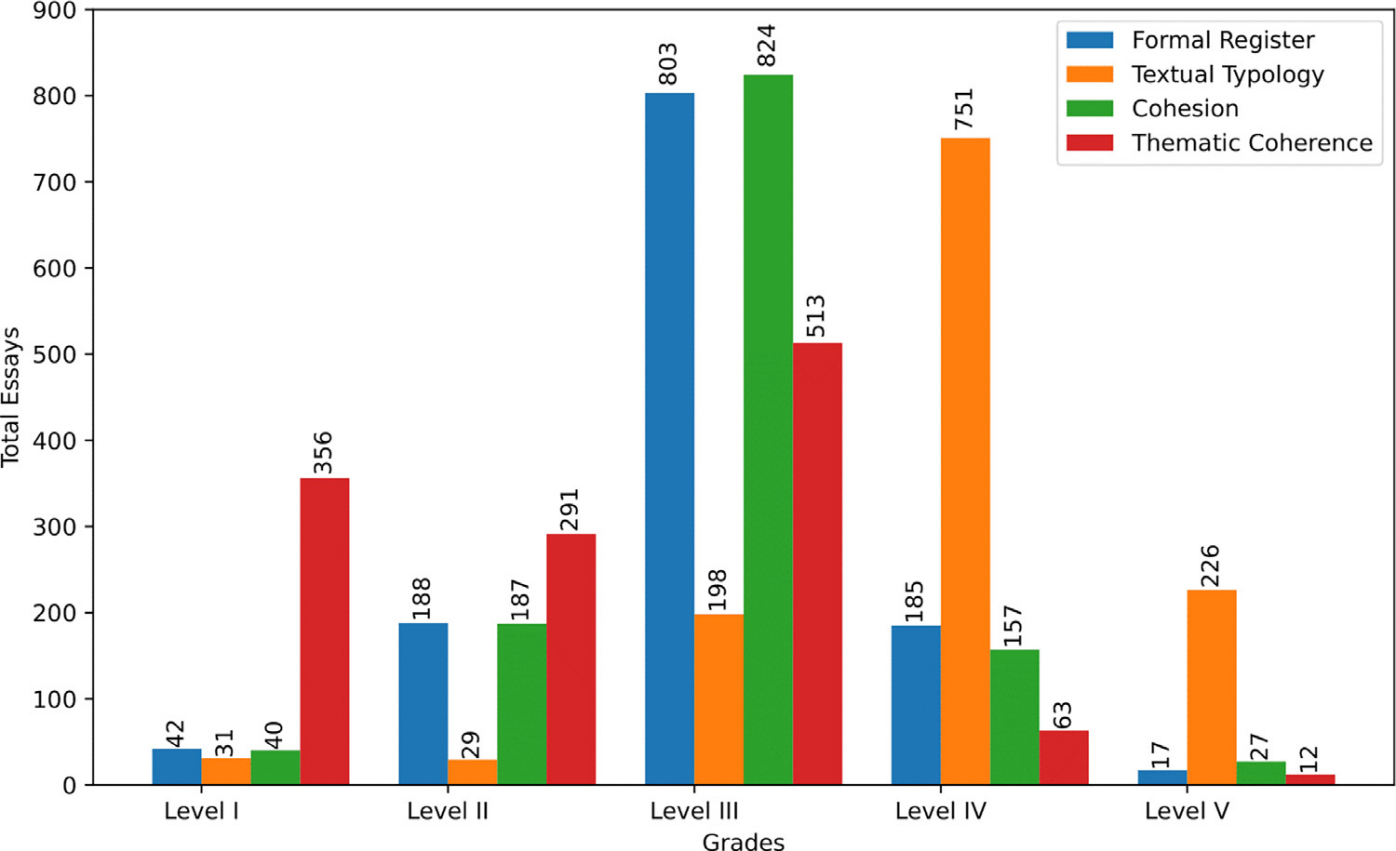
Essays distribution by level for each assessed competency on the complete corpus.

For each of the four competencies, and following the criteria outlined in Table 4, human evaluators assigned a proficiency level ranging from I (1) to V (5), where Level I indicates a complete lack of knowledge in the competency domain, and Level V represents full mastery. The evaluators were linguistically trained teachers with experience in assessing this type of textual production. Over twelve weeks, each evaluator independently assigned scores to each competency. Due to the inherent complexity and subjectivity of this evaluation process, disagreements among annotators were frequent. To address this issue, a third, more experienced evaluator was included to resolve discrepancies between the initial two annotators. Fig. 2 presents the distribution of essays across the corpus by proficiency level for each evaluated competency.

**Fig. 3:**
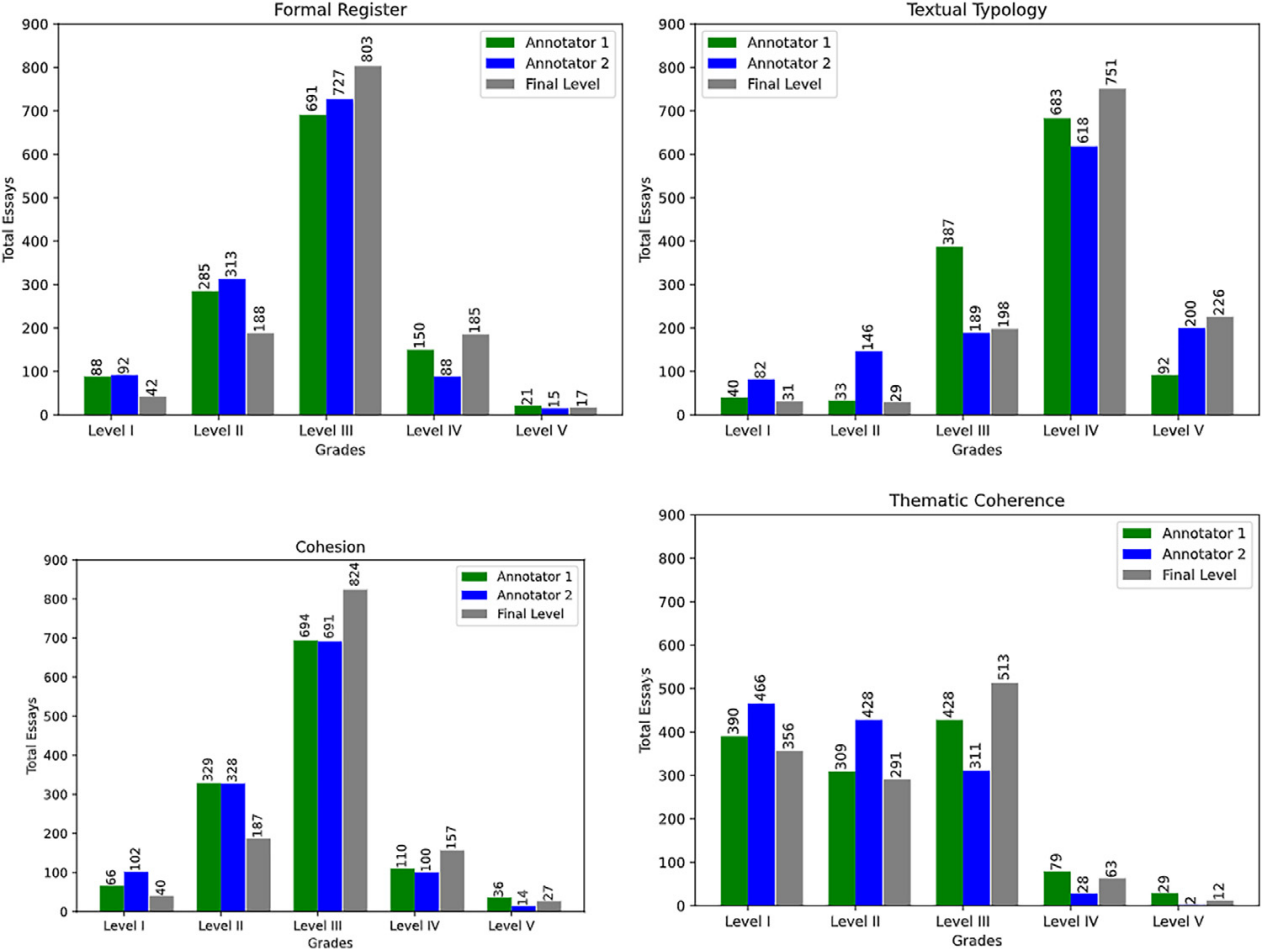
Total number of essays based on the grades assigned by the first two annotators and the final annotator in each assessed competency.

A significant imbalance can be observed between the number of essays by level and competence. For instance, there are generally few essays at levels I and V, with most of the essays concentrated at level III. This scenario reflects the reality of evaluations carried out by human evaluators, i.e., it is unusual to have students who demonstrate a total lack of ability in a specific competency, and only some demonstrate complete mastery. Fig. 3 shows the distribution of the number of essays in each competency based on the grades assigned by the first two annotators and the final grade after the divergency resolution.

**Fig. 4:**
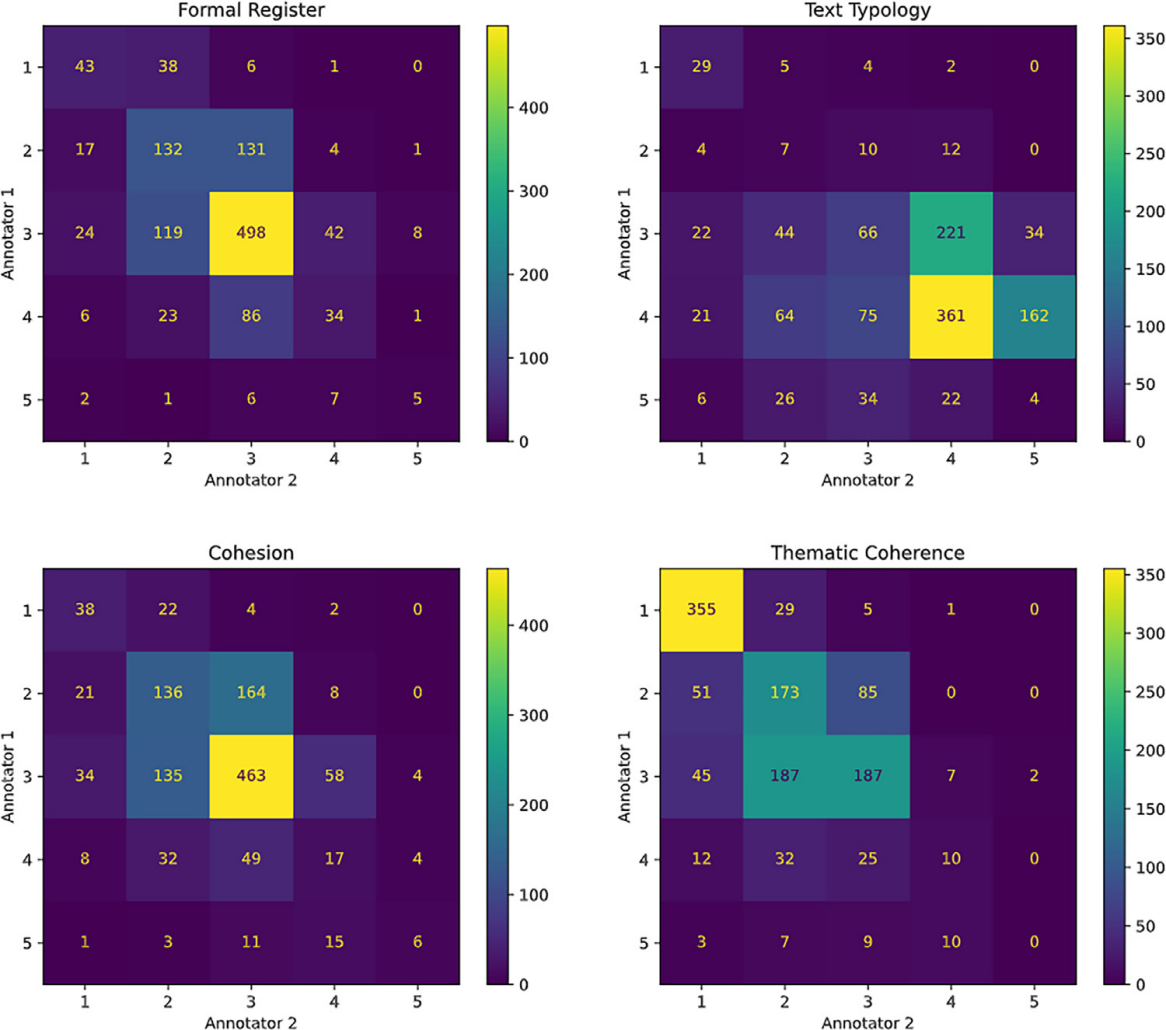
Confusion matrices showing agreement and disagreement scenarios between the first two annotators.

The average Cohen's Kappa agreement score between annotators 1 and 2 across the four competencies was 0.2475. The overall average agreement between the first and third annotators and between the second and third annotators was 0.5405 and 0.5650, respectively. Despite the low agreement level between the first two annotators, most disagreements occurred at adjacent levels. For example, one annotator might have assigned a level IV to an essay, while the other assigned level III or V. Such variations are expected in writing assessments due to the complexity and subjectivity of the evaluation process. To illustrate this pattern, Fig. 4 displays the confusion matrices, highlighting agreement and disagreement cases between the first two annotators.

Except for the textual typology competency, which presented the highest level of disagreement, it is possible to observe a higher occurrence of concordant items (main diagonal) in the other competencies and that most divergences are at neighboring levels. This observation emphasizes the complexity of the task, even for trained professionals, and highlights the challenge of developing computer systems to automate it.

## Limitations

A noteworthy limitation of the present database version is its size, consisting of 1235 essays, which is deemed relatively small, particularly for employing deep neural network models. It is worth noting that the annotation process was conducted manually, demanding considerable time and effort. Consequently, scaling this process to augment the database remains a challenging task.

## Ethics Statement

The proposed dataset does not involve animal experiments or data collected on social media platforms. All essays collected were anonymized, and no personal data of the students was used.

## Credit Author Statement

**Hilário Oliveira**: Methodology, Data curation, Writing – original draft, reviewing and editing; **Rafael Ferreira Mello**: Conceptualization, Methodology, Data curation, Writing - reviewing and editing; **Péricles Miranda**: Methodology, Writing - reviewing and editing; **Hyan Batista**: Data curation, Software; **Moésio Wenceslau da Silva Filho**: Data curation, Software; **Thiago Cordeiro**: Conceptualization, Supervision; **Ig Ibert Bittencourt**: Conceptualization, Supervision; **Seiji Isotan**i: Conceptualization, Supervision.

## Data Availability

KaggleBrazilian Portuguese Narrative Essays Dataset (Original data).

## Footnotes

1 https://spacy.io/.
